# Influence of Natural Fractures and Laminae on Fracture Propagation and Failure Mode of Continental Shale

**DOI:** 10.3390/ma17184655

**Published:** 2024-09-23

**Authors:** Beixiu Huang, Sijia Qiao, Lihui Li, Xiangbo Gao, Xiao Li, Pathegama Gamage Ranjith

**Affiliations:** 1 State Key Laboratory of Lithospheric and Environmental Coevolution, Institute of Geology and Geophysics, Chinese Academy of Sciences, Beijing 100029, China; huangbeixiu@mail.iggcas.ac.cn (B.H.); qiaosijia22@mails.ucas.ac.cn (S.Q.); 2 Innovation Academy for Earth Science, Chinese Academy of Sciences, Beijing 100029, China; lixiao@mail.iggcas.ac.cn; 3 College of Earth and Planetary Sciences, University of Chinese Academy of Sciences, Beijing 100049, China; 4 Key Laboratory of Coastal Environment and Resources of Zhejiang Province, School of Engineering, Westlake University, Hangzhou 310030, China; gaoxiangbo@westlake.edu.cn; 5 Key Laboratory of Deep Petroleum Intelligent Exploration and Development, Institute of Geology and Geophysics, Chinese Academy of Sciences, Beijing 100029, China; 6 Deep Earth Energy Laboratory, Department of Civil Engineering, Monash University, Melbourne 3800, Australia; ranjith.pg@monash.edu

**Keywords:** continental shale, natural fracture, soft-to-hard lamina, fracture propagation, failure mode

## Abstract

Natural fractures and laminae are well-developed in continental shale, which greatly affects the fracture propagation and failure mode. Based on the natural fractures and laminae developed in the outcrops of Triassic continental shale from the southern Ordos Basin, China, four different types of shale models are constructed in this research. The CASRock software V1.0 is utilized to conduct numerical simulations to investigate the influence of natural fractures and soft-to-hard laminae on the mechanical behavior of continental shale. The results demonstrate that the uniaxial compressive strength of shale models can improve by up to 34.48% when soft-to-hard laminae are present, but it can drop by up to 18.97% when weak interfaces are present. New fractures are consistently initiated at the ends of natural fractures, with various propagation patterns in different laminae. Fractures in soft laminae usually propagate in an oblique path at an angle *β* ≈ 20°–30° relative to the direction of compressive stress, manifesting as shear fractures. Fractures in medium-to-hard laminae tend to propagate parallel to compressive stress, primarily featuring tensile fractures. The ultimate fracture morphology becomes more complex as soft, medium, and hard laminae and weak interfaces occur successively. It changes from a nearly linear fracture to an echelon pattern with more secondary fractures and finally a network shape, with a total fracture area increase of up to 270.12%. This study reveals the combined effect of natural fractures, soft-to-hard laminae, and weak interfaces on the fracture propagation and failure model of continental shale, providing support for fracturing optimization based on shale’s authentic structure characteristics.

## 1. Introduction

Oil and gas are currently the most important primary energy sources globally. They are anticipated to remain the dominant contributors to world energy consumption over the next two decades [1,2], before achieving carbon neutrality by 2050 [3]. Since the United States has made a breakthrough in the core technology of shale gas development, a vigorous “shale revolution” has been set off worldwide [4]. Different from marine shale, continental shale is deposited in the semi-deep-water to the deep-water lacustrine sedimentary environment, presenting strong heterogeneity [5,6]. Outcrop observation and core analysis show that continental shale is well-developed with diverse laminae, such as organic-rich lamina, clay lamina, silty lamina, tuffaceous lamina, and pyrite lamina, forming various combinations [6,7,8]. These laminae may store free gas and help transport natural gas desorption from organic matter to the wellbore [9,10], enhancing gas-bearing and fracturing properties [7]. Additionally, fractures are integral to shale’s geological structure, with numerous fractures developing between laminae or beddings [11,12,13,14]. These fractures are both oil and gas reservoir spaces and seepage channels. They can significantly aggravate the anisotropy of the reservoir [15,16,17].

The influence of laminae or beddings on the mechanical behavior of shale has been extensively studied based on experimental tests [18,19,20,21]. For instance, Zou et al. [22] studied the internal fracture morphology of Triassic continental shale in a true triaxial test with industrial X-ray computed tomography (CT), addressing that partially opened bedding planes are helpful in increasing fracture complexity. Yang et al. [23] revealed two types of fracturing fluid immersion effect (lubrication-dominated and softening-dominated) on the mechanical properties and failure mechanism of Jurassic continental shale. Zhao et al. [24] compared the mechanical behavior of Triassic continental shale and concluded that laminated shale had lower compressive strength and stronger anisotropy than bedding shale, with more natural microfractures. Huang et al. [25] investigated the meso-mechanical anisotropy and fracture propagation of Triassic continental shale with a stereomicroscope and micro-CT. Zou et al. [26] conducted a series of comprehensive laboratory sand-fracturing experiments and fracturing conductivity tests on Permian continental shale, pointing out that the hydraulic fracture growth path was likely to be deflected and bifurcated by the natural fractures laterally and by the oblique laminae vertically. These studies enriched our understanding of the mechanical behavior of continental shale deposited in different geological ages.

Due to continental shale’s developed laminae and natural fractures, it is challenging to prepare adequate samples for investigating its mechanical behavior under various conditions [27]. Numerical simulation is a valuable alternative [28] and has been widely used to characterize fracture propagation and fracture patterns in shale [29,30,31,32], including the spatial heterogeneity and material anisotropy effects [18,33], the interaction of lamination/interface, natural fractures, and hydraulic fractures [34,35]. Sesetty and Ghassemi [36] studied hydraulic fracture propagation in naturally fractured rock at a large scale, highlighting that fluid diversion into natural fracture can initiate wing fracture growth. Zhou et al. [37] numerically revealed the distribution law of hydraulic fractures in shale with different weak bedding plane densities, bedding strength, and fracturing engineering parameters. Suo et al. [38] investigated the influence of thickness ratio, combination form, and confining pressure of continental sandstone–shale on the mechanical properties of composite rock samples. These studies enhance our understanding of the individual effect of bedding and natural fractures on the mechanical properties of shale. However, the shale models used in these studies usually consist of simplified homogeneous laminae or beddings [13,39], which are different from real laminae developed in shale reservoirs and may result in large errors. Additionally, the combined effect of natural fractures and laminae on the mechanical behavior of continental shale has rarely been studied and remains unclear.

In this paper, we first constructed shale models with different geological structures and set mechanical parameters for different laminae based on prior experimental results. The models were based on real natural fractures and soft-to-hard laminae developed in continental shale reservoirs. Then, we used a cellular automata software for engineering rockmass fracturing process (i.e., CASRock V1.0) to numerically simulate the mechanical behavior of various shale models. To investigate the combined effect of natural fractures and various laminae on the mechanical behavior of shale, the stress–strain curves, acoustic emission (AE) events, fracture propagation process, and failure modes of various shale models were obtained and compared. The total fracture areas were further quantified to explore the fracture complexity of continental shale influenced by various structure features.

## 2. Model and Numerical Simulation Scheme

### 2.1. CASRock Introduction

The numerical methods in rock mechanics usually derive a numerical approximation of the global equations in a “top-down” manner [40], which is not suitable for addressing the local behavior evolution of rock. Pan et al. [41] introduced cellular automata (CA) theory to rock mechanics and developed a cellular automata software for engineering rockmass fracturing process, namely CASRock [42], by utilizing a “down-top” method. A cellular automaton is composed of a cell, a cell space, a cell state, a neighborhood, and updating rules. According to the CA localization theory, the state of a cell depends on the states of itself and its neighbors, which is described by the CA updating rule [43,44]. For an elasto-plastic mechanical process, based on the stress equilibrium equation, geometrical equation, constitutive equation, and yield criterion, the equilibrium equation of the cell can be written as Equation (1).

(1) $$K_{i}∆u_{i}=∆F_{i}+∆F_{i}^{p}$$

where $K_{i}$, $u_{i}$, $F_{i}$, and $F_{i}^{p}$ are the cell stiffness, cell node displacements, cell nodal force, and the equivalent nodal force induced by plasticity, respectively.

The neighboring cell nodal forces can be obtained by Equation (2):

(2) $$\left\{∆F_{i}^{k}\right\}=\left[K_{i}^{j}\right]\cdot \left\{∆u_{i}^{j}\right\}$$

where $∆F_{i}^{k}$, $K_{i}^{j}$, and $∆u_{i}^{j}$ are the neighboring cell nodal force, cell element stiffness, and cell nodal displacement, respectively.

The change in force can induce a change in displacement and vice versa. This process will occur among cells in the system. The system will maintain its static equilibrium state when the self-organization phenomenon of $∆u_{i}\to$ 0, $∆F_{i}^{k}\to$ 0 appears.

The self-organizing expansion of discontinuous fractures is realized by fracture path recognition based on local information, avoiding treating the overall structure as a partial differential equation edge value problem and solving large linear equations. Additionally, the software embedded rock fracture degree (*RFD*) acts as an index for the fracture measurement of the local area or a single simulated cell [45]. It facilitates the description of the fracture process and engineering rock mass stability. Therefore, we conducted numerical simulations with CASRock to emphasize the influence of natural fractures and laminae on the fracture propagation of shale. The embedded *RFD* can be calculated with Equation (3).

(3) $$\mathrm{RFD}=\left\{\begin{array}{c} 1-\frac{g\left(\theta \right)\sqrt{Ap^{2}+\mathrm{Bp}+C}-q}{g\left(\theta \right)\sqrt{Ap^{2}+\mathrm{Bp}+C}},prepeak\\ 1+\frac{\varepsilon_{V}^{p}}{{(\varepsilon }_{V}^{p}){|}_{\mathrm{limit}}}+\frac{\bar{\gamma }}{(\bar{\gamma }){|}_{\mathrm{limit}}},postpeak \end{array}\right.$$

where $g\left(\theta \right)$ is the shape function of the Lode angle *θ* in the deteriorating plane, p is the mean principal stress, and q is the equivalent shear stress. Supposing that the *RFD* of the pre-peak stage follows a modified Wiebols and Cook failure criterion [46], which can reflect the strain energy accumulation induced by micro-cracking, *A*, *B*, and *C* are the correlation coefficients of the Wiebols Cook criteria. $\varepsilon_{V}^{p}$ is the plastic volume strain, and $\bar{\gamma }$ is the equivalent plastic shear strain, while ${(\varepsilon }_{V}^{p}){|}_{\mathrm{limit}}$ and $(\bar{\gamma }){|}_{\mathrm{limit}}$ are their corresponding limit values. The *RFD* in the post-peak phase describes the rupture development caused by changes in volume and shape.

The *RFD* range is of 0–2, where *RFD* < 1 indicates the degree of rupture development in the pre-peak phase, and *RFD* ≥ 1 characterizes the degree of rupture development in the peak or post-peak phase. *RFD* > 2 indicates being at the residual stress level. For visualization of the fracture propagation characteristics, *RFD* values greater than 2 are set to 2.

Furthermore, acoustic emission (AE) signals effectively monitor the brittle failure process of rocks [47]. In quasi-brittle materials like rocks, AE signals are mainly related to the release of elastic energy [48]. Under the assumption that AE count is proportional to the number of destructive elements and that the strain energy of these destructive elements is released in the form of acoustic emission, AE count can be determined according to the number of destructive elements. The total energy release is then calculated from the strain energy released by these elements [49].

### 2.2. Shale Modeling

**Figure 1:**
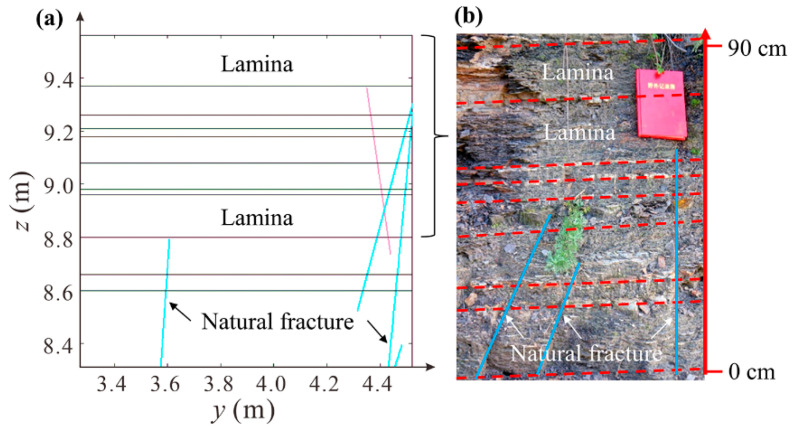
A two-dimensional rock mass structure model (**a**) of natural fractures (indicated by blue and pink oblique lines) and laminae in continental shale established by Monte-Carlo simulation and typical outcrop photo (**b**) [12].

To closely resemble the real rock mass structure of a continental shale reservoir, we generated numerical models of shale based on a two-dimensional rock mass structure model of continental shale we have previously published (Figure 1a). The structure model was generated by Monte-Carlo simulation, based on statistical structural parameters of natural fractures and laminae we have identified in the real continental shale outcrops (Figure 1b) [8,12]. The continental shale was deposited during the late Triassic, belonging to the Yanchang Formation, Ordos Basin, China, acting as high-quality hydrocarbon source rocks for shale oil and gas exploitation.

To distinguish the combined influence of natural fractures and various laminae, four shale models with increasingly complex structures were developed. Specifically, all the shale models consisted of the same five natural fractures, with lamina heterogeneity increased from one type (soft laminae) to two types (soft-to-medium laminae) to three types (soft-to-medium-to-hard laminae). Additionally, two weak interfaces were added in the last model to resemble the authentic complex geological structure of continental shale. The detailed components of the four models are as follows.

Case_1 was composed of five natural fractures and one type of lamina (soft laminae); Case_2 was composed of five natural fractures and two types of laminae (soft-to-medium laminae); Case_3 was composed of five natural fractures and three types of laminae (soft-to-medium-to-hard laminae); Case_4 was composed of five natural fractures, three types of laminae (soft-to-medium-to-hard laminae), and two weak interfaces.

**Figure 2:**
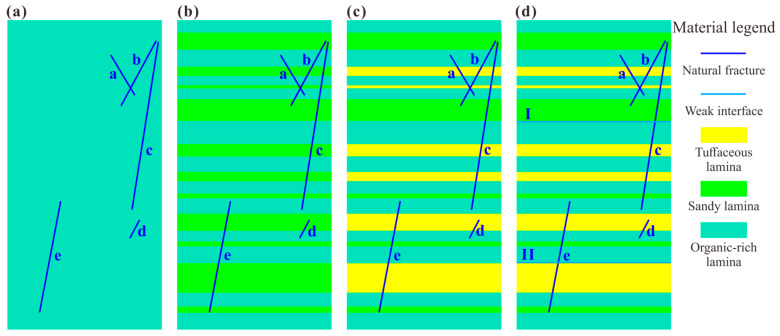
Rock mass structure models of shale developed with natural fractures and various laminae. (**a**–**d**) Correspond to Case_1, Case_2, Case_3, and Case_4, respectively. Note that the blue oblique lines with lowercase letters “a–e” represent natural fractures NF_a to NF_e. The light blue horizontal lines with numbers “I, II” indicate weak interfaces WI_I and WI_II.

According to the multi-scale mechanical experiments on different laminae [25,50,51], the organic-rich laminae were characterized as soft, displaying an elastic modulus of approximately 15 GPa. In contrast, the sandy laminae exhibited a moderate elastic modulus of around 43 GPa, while the pyritic and tuffaceous laminae demonstrated a hard nature with a large elastic modulus of about 120 GPa. Therefore, we took the organic-rich laminae, sandy laminae, and tuffaceous laminae as the soft, medium, and hard laminae, respectively. The five natural fractures were labeled as NF_a to NF_e, and the two weak interfaces were denoted as WI_I and WI_II, as shown in Figure 2.

The model dimensions were 50 cm in diameter and 100 cm in height, with mesh elements divided into squares with a side length of 5 mm, resulting in a total of 20,000 cells and 20,301 nodes.

### 2.3. Model Parameters

The input mechanical parameters of various materials in the models are presented in Table 1, including elastic modulus *E*, Poisson’s ratio *v*, initial cohesion $c_{0}$, residual cohesion $c_{r}$, initial internal friction angle $\varphi_{0}$, residual internal friction angle $\varphi_{r}$, initial tensile strength $\tau_{0}$, and residual tensile strength $\tau_{r}$. Considering that the mineral components of the shale laminae are heterogeneous to some extent, the organic-rich laminae, sandy laminae, and tuffaceous laminae are regarded as heterogeneous models. The Weibull’s distribution is used to assign elastic modulus and strength parameters to the cellular units, with a homogeneity coefficient of 3 and a random seed number of 10. The natural fracture and weak interface components are considered relatively simple and homogeneous models.

Shale exhibits strong brittleness, and this brittleness is reflected by the brittle behavior of cellular units. For laminae and weak interfaces, a brittle constitutive relationship was adopted. Natural fractures, having much lower strength compared to laminae, were simplified with weakened cellular units, assumed to follow the ideal plastic-constitutive relationship. The Mohr–Coulomb with tension cut-off intensity criterion was utilized to determine whether the cellular unit yielded. The boundary condition involved bidirectional uniaxial compression with a loading rate of 10^−6^ m/s. The simulation was conducted using a planar strain model and the calculation was stopped when the stress–strain curve reached a stable residual stage.

## 3. Results

### 3.1. Stress–Strain Curves and AE Events

**Figure 3:**
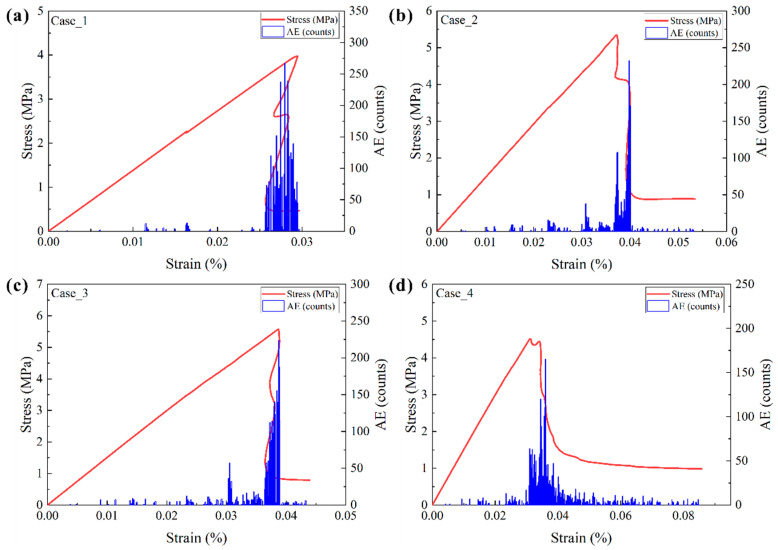
Stress–strain-AE curves of shale with various structural models. (**a**–**d**) Correspond to Case_1, Case_2, Case_3, and Case_4, respectively.

The stress–strain curves and AE events for shale with various structures are presented in Figure 3. They indicated significant variations in stress–strain curves and AE events with laminae type. For the shale comprising organic-rich laminae and natural fractures (Case_1), AE events were scarce when stress was below the peak. Numerous AE events concentrated in the post-peak stage (Figure 3a), reaching a maximum of 267 counts per step, suggesting a pronounced brittle failure. In the shale model consisting of organic-rich laminae, sandy laminae, and natural fractures (Case_2), intermittent AE events occurred in the elastic stage. After reaching peak stress, the stress–strain curve exhibited two nearly linear drops. The first drop was small, accompanied by some AE events, while a large amount of AE events occurred in the second drop (Figure 3b), peaking at 232 counts per step.

For the shale model containing three types of laminae and natural fractures (Case_3), intermittent AE events were observed in the elastic stage, with the majority concentrated during stress drop in the post-peak stage. The stress drop exhibited a concave-then-convex curve shape (Figure 3c). AE events increased with strain, and the maximum AE event was comparable to Case_2, reaching 224 counts per step. Regarding the shale model consisting of three types of laminae, weak interfaces, and natural fractures (Case_4), AE events (Figure 3d) were weak in the linear stage but increased slightly during the stress yield stage. The stress then dropped nearly linearly, followed by a plastic failure. The maximum AE events for this model were much lower than the other models, with a value of about 165 counts/step. This suggested that the fracture propagation in this model was much gentler than in other models.

**Figure 4:**
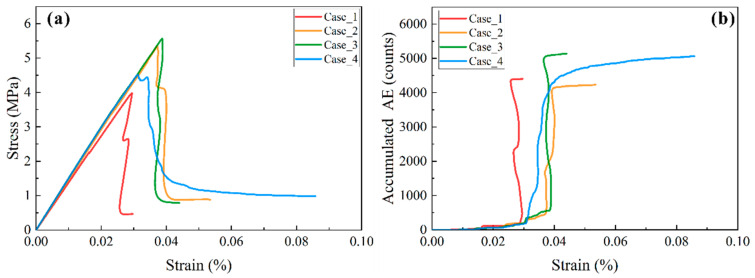
Comparison of stress–strain (**a**) and accumulated AE–strain curves (**b**) of shale with various structures.

Figure 4a presents the stress–strain curves for the four models. The slopes of these curves indicate that the elastic modulus of Case_1 was the lowest, while the elastic modulus of Case_2, Case_3, and Case_4 was almost identical, in the approximate range of 14.37–14.38 GPa, just slightly higher than that of Case_1 (~13.52 GPa). This suggested that lamina type and a few weak interfaces had little influence on the elastic modulus of shale. The peak strength of Case_3 was the highest (~5.57 MPa), with a decreasing order of Case_2, Case_4, and Case_1, having a value of 5.35 MPa, 4.51 MPa, and 3.98 MPa, respectively. The largest increase ratio of uniaxial compressive strength of Case_3 relative to Case_1 was 34.48%. This indicated that the occurrence of medium-to-hard laminae, such as sandy laminae and tuffaceous laminae, would significantly increase the peak strength of whole shale. However, even though only two weak interfaces occurred in the shale with soft-to-hard laminae, its peak strength decreased remarkably, with a decreasing ratio of uniaxial compressive strength of Case_4 relative to Case_3 of 18.97%.

The accumulated AE–strain curves in Figure 4b show that the accumulated AE events for the four models were in a range of 4000–5200 counts. Case_3 had the most AE counts, closely followed by Case_4, with Case_1 having slightly larger AE events than Case_2. The differences between Case_1 and Case_2 suggested that the medium lamina would increase the axial strain of the model and decrease the released elastic energy. The approximate accumulated AE counts of Case_3 and Case_4 demonstrated that weak interfaces had a small influence on elastic energy release but a significant impact on failure mode.

### 3.2. Fracture Propagation Characteristics

**Figure 5:**
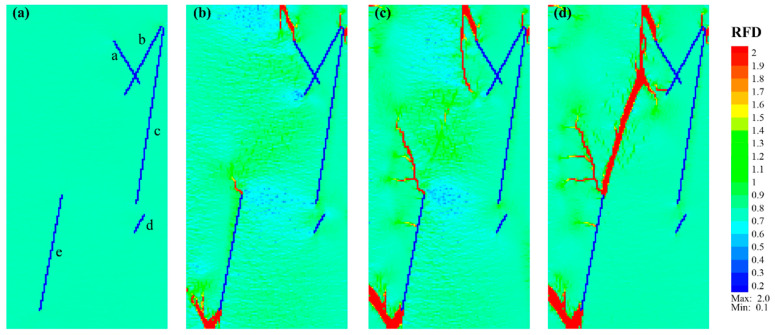
Fracture propagation characteristics of shale model Case_1 consisting of natural fractures (blue lines with lowercase letters “a–e”) and soft laminae (light green matrix). (**a**–**d**) Correspond to the *RFD* cloud diagram of Step 0, Step 192, Step 231, and Step 294, respectively.

To investigate the fracture initiation, propagation, and connection in shale models with various structures, representative *RFD* cloud diagrams were selected for the study. Figure 5 shows the fracture propagation of Case_1 under uniaxial compression conditions. The *RFD* cloud diagrams of Case_1 at Step 0 (beginning) and Step 192 (Figure 5a,b) indicated that those new fractures mainly initiated from the ends of pre-existing fractures NF_a and NF_e. The fracture originating from the upper end of NF_a first extended to the top of the model and then propagated downward from the top to the center along the direction of maximum stress. Simultaneously, the fracture initiating from the lower end of NF_e initially extended to the bottom, following a nearly parallel direction to NF_e, and then propagated upward at an angle *β* ≈ 35° (herein, *β* refers to the angle between the fracture direction and the direction of the maximum compressive stress, with a range of 0–90°), reaching the left interface of the model. The fracture initiating from the upper end of NF_e propagated upward in a direction of *β* ≈ 22°, accompanied by horizontal branch fractures.

As Case_1 progressed to Step 231 (Figure 5c), new fractures formed in a direction of *β* ≈ 18° in the near-middle position between the upper and lower fractures. Subsequently, these fractures propagated toward both ends in a nearly straight manner. Finally, the new fractures at the upper and lower ends connected, forming a single main fracture, as shown in Figure 5d. Some additional fractures occurred near the upper end of NF_e, but their propagation was not pronounced. With continuous loading, small branch fractures developed around the main fracture. A new fracture was initiated near the lower end of NF_b, expanding horizontally and gradually approaching the branch fractures of the main fracture. Notably, no visible fracture was initiated from NF_d during the entire loading, possibly due to its relatively short length and considerable distance from the main fracture, resulting in minimal impact on surrounding units.

**Figure 6:**
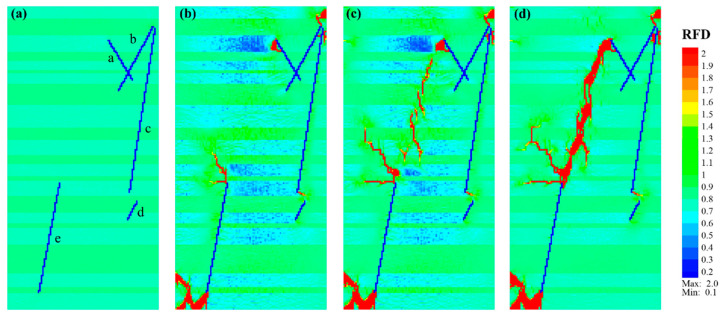
Fracture propagation characteristics of shale model Case_2 consisting of natural fractures (blue lines with lowercase letters “a–e”) and soft-to-medium laminae (light green to green layers). (**a**–**d**) Correspond to the *RFD* cloud diagrams of Step 0, Step 183, Step 261, and Step 318, respectively.

The fracture propagation of Case_2 is presented in Figure 6. Similar to Case_1, new fractures are predominantly initiated from the ends of pre-existing natural fractures (NF_a, NF_b, NF_c, and NF_e). However, unlike Case_1, where the new fractures primarily extended to the top or bottom, the fracture propagation in Case_2 was notably affected by the presence of medium sandy laminae (depicted in green). Take the NF_a as an example, whose upper end is located in an organic-rich lamina (light green) and overlaid by a medium sandy lamina. New fractures were initiated at the upper end and extended as clusters within the organic-rich lamina, without extending toward the top (Figure 6a,b). The fracture initiating at the upper end of NF_b first obliquely crossed the sandy lamina and then expanded into the organic lamina at the top. Similarly, the fracture originating at the lower end of NF_e mainly extended within the organic-rich lamina, with a direction of *β* ≈ 42° before vertically propagating into the sandy lamina.

The fracture initiated at the upper end of NF_e vertically crossed the overlaid sandy lamina and entered the organic lamina. Initially, it extended horizontally for a short distance, and then obliquely propagated upward to the sandy lamina before vertically crossing it (Figure 6c). There was one fracture initiated from the lower end of NF_c, but its subsequent expansion was not pronounced. As the loading continued, a group of echelon fractures formed in the middle area between the upper ends of NF_e and NF_a. They gradually extended to both ends and eventually connected, forming a sausage-like main fracture with *β* ≈ 21° (Figure 6d). Generally, fractures in this model exhibited greater complexity than those in Case_1, showing horizontal propagation (*β* ≈ 90°) in the organic-rich lamina, oblique propagation (*β* ≈ 20–40°) to the upper sandy lamina, and nearly vertical crossing (*β* ≈ 0°) in sandy lamina.

**Figure 7:**
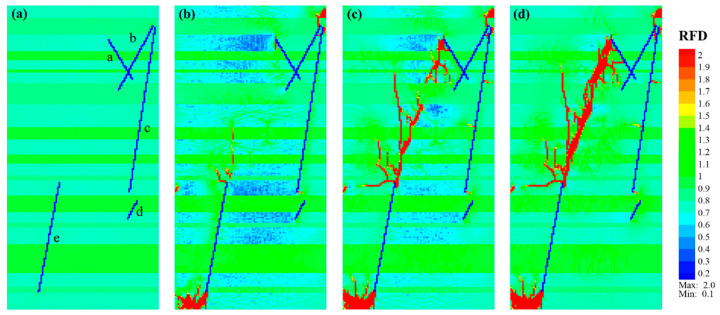
Fracture propagation characteristic of shale model Case_3 consisting of natural fractures (blue lines with lowercase letters “a–e”) and soft-to-medium-to-hard laminae (light green to green to bright green layers). (**a**–**d**) Correspond to the *RFD* cloud diagrams of Step 0, Step 174, Step 207, and Step 270, respectively.

For Case_3, its fracture characteristics had some similarities to Case_2. When the fracture encountered medium-to-hard laminae, such as sandy laminae (green) or tuffaceous laminae (bright green), they mainly propagated vertically through these laminae. However, in the organic-rich laminae (light green), they tended to expand obliquely (Figure 7). In contrast to Case_2, the vertical crossing-lamina fractures were more remarkable in Case_3, and the fracture area also increased significantly. A new fracture was initiated vertically at the upper end of NF_a and mainly expanded downward with branches.

Some fractures initiating at the upper end of NF_e first extended over a short distance along the interface between the organic-rich lamina and sandy lamina, and then vertically passed through the overlaid thin sandy lamina (Figure 7a,b). After reaching the organic-rich lamina, they extended upward with an arc shape (Figure 7c). Simultaneously, at the left boundary of the organic-rich lamina where the upper end of NF_e was located, some new fractures developed in a direction of *β* ≈ 35°, but their later expansion was relatively slow. The fractures initiated at the lower end of fracture NF_e mainly extended within the organic-rich lamina, with some fractures in the later stage extending vertically upward through overlaid sandy laminae. As the loading continued, a group of echelon fractures gradually appeared in the middle of the model, finally connecting with new fractures in the upper and lower parts to form a main fracture with a strike of *β* ≈ 23° (Figure 7d). There was also one new fracture initiated from the middle of NF_a, which extended horizontally and connected with the main fracture.

**Figure 8:**
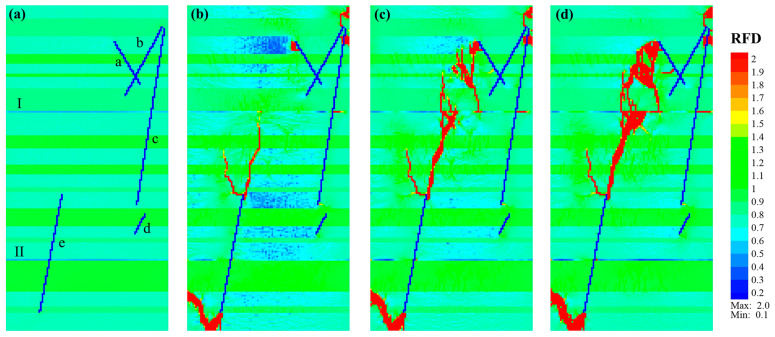
Fracture propagation characteristic of shale model Case_4 consisting of natural fractures (blue lines with lowercase letters “a–e”), soft-to-medium-to-hard laminae (light green to green to bright green layers), and two weak interfaces (light blue lines with numbers “I, II”). (**a**–**d**) Correspond to the *RFD* cloud diagrams of Step 0, Step 162, Step 204, and Step 474, respectively.

Case_4 was mainly characterized by fractures crossing the laminae, with a few fractures extending horizontally around the middle of WI_I and NF_a. Figure 8a,b shows that new fractures are mainly initiated along the upper ends of NF_a and NF_b, as well as the upper and lower ends of NF_e. The fracture initiated at the upper end of fracture NF_b was similar to Case_3, which extended toward the right boundary in an organic-rich lamina after vertically crossing a medium sandy lamina. Some new fractures occurred near the upper end of NF_c and were concentrated in the organic-rich lamina. The morphology of the fractures initiating at the lower end of fracture NF_e was similar to that in Case_1 and Case_2. The fracture first extended toward the bottom, then to the upper right, with an angle of *β* ≈ 30° and *β* ≈ 0° (vertical) in the organic-rich laminae and sandy laminae, respectively. The upward propagation of the fracture initiated at the upper end of fracture NF_e was similar to Case_ 3, mainly with a fracture passing through the sandy and tuffaceous laminae almost vertically and extending obliquely in the organic-rich laminae (Figure 8c).

However, compared with Case_3, there were no horizontally propagated long-branched fractures initiating at the upper end of NF_e. This is probably due to the occurrence of weak interfaces. Along the upper extension of NF_e, fractures vertically passed through the medium-to-hard laminae, and fractures which obliquely crossed the organic-rich laminae appeared successively and connected. When the fractures expanded to the organic-rich lamina below WI_I, one part passed through WI_I and continued to expand vertically upward, while the other part crossed the organic-rich lamina obliquely and connected with the weak interface. Then, a triangular region of fractures was formed, and denser micro-fractures were generated with continuous loading (Figure 8d).

Additionally, the propagations of fracture initiating from the upper end of NF_a in Case_3 and Case_4 were different. The former mainly extended downwards like a tree after a vertical extension. While the latter tended to propagate downward vertically first, it then propagated to both ends along a direction of *β* ≈ 35°. New vertical and oblique fractures are constantly generated during the upward expansion process. Concerning the downward expansion, it occurred in a stepped manner until connecting with the weak interface. With continuous loading, the downward propagating fractures initiated at the upper end of NF_a, and the upward propagating fractures initiated at the upper end of NF_e, connected, and formed a main fracture (Figure 8d), with the angle between the main fracture and the direction of maximum compressive stress being *β* ≈ 18°. 

### 3.3. Failure Mode Features

The aforementioned fracture propagation characteristics highlight the significant influence of natural fractures, lamina type, and weak interfaces on the mechanical behavior of shale. To analyze the failure mode features, we generated equivalent plastic shear strain (Epstn) and equivalent plastic tensile strain (Epttn) cloud maps corresponding to the final *RFD* cloud maps of Case_1 to Case_4 for comparative studies.

**Figure 9:**
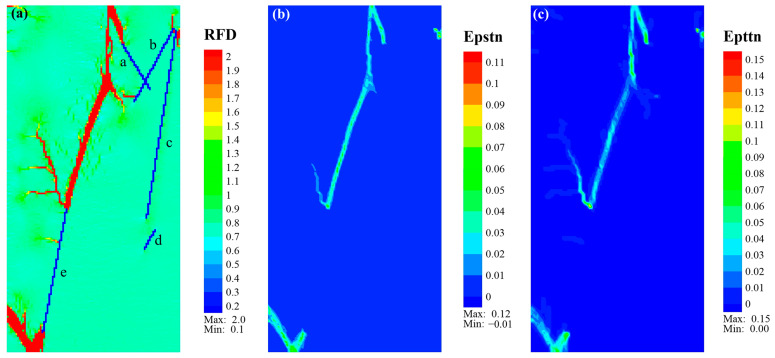
Eventual cloud diagram of *RFD* (**a**), Epstn (**b**), and Epttn (**c**) of Case_1. Note that the lowercase letters “a–e” represent natural fractures NF_a to NF_e.

Figure 9 shows the final *RFD* cloud map and strain cloud map for Case_1. The ultimate fracture morphology of shale with only organic-rich laminae was relatively simple, with an approximate width along the main fracture. The main fracture in the middle of the model was primarily shear, while the fractures at the upper and lower ends of the main fracture were dominated by tensile fracture. The fractures that initiated from the upper ends of NF_a to the top and from the lower end of NF_e to the bottom boundary exhibited a shear–tensile composite failure, similar to the fractures initiated along the upper ends of NF_b and NF_c. The fracture at the bottom boundary extended toward the left boundary with predominant shear strain.

**Figure 10:**
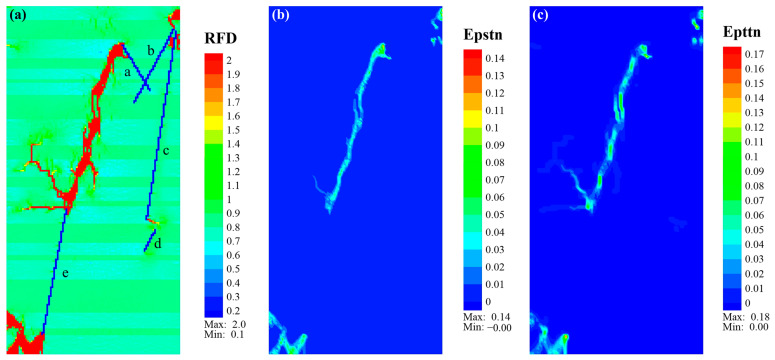
Eventual cloud diagram of *RFD* (**a**), Epstn (**b**), and Epttn (**c**) of Case_2. Note that the lowercase letters “a–e” represent natural fractures NF_a to NF_e.

In Case_2, the main fracture morphology was an echelon fracture (Figure 10a). The fractures in organic-rich laminae were relatively wider than those in sandy laminae. They presented several nearly parallel vertical fractures in sandy laminae connected to oblique fracture clusters in organic-rich laminae. The fractures in the organic-rich laminae mainly exhibited shear failure (Figure 10b), while the fractures in the sandy laminae mainly exhibited near-vertical tensile fracture, as shown in Figure 10c. The branch fractures that extended from the upper end of NF_e to the upper left and from the upper end of NF_b to the right boundary also presented tensile fractures. The fracture around the lower end of NF_e was similar to Case_1, predominated by a tensile fracture with local shear fracture.

**Figure 11:**
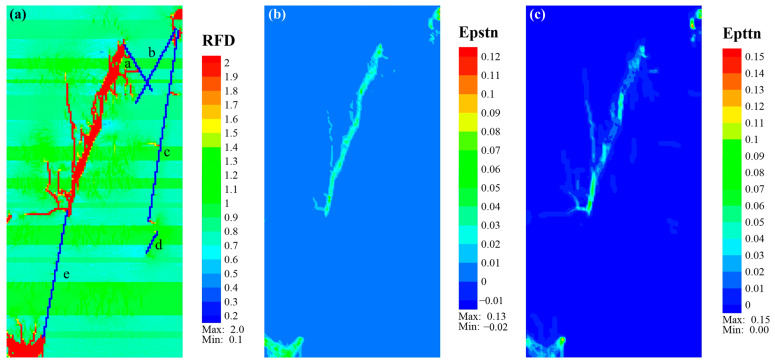
Eventual cloud diagram of *RFD* (**a**), Epstn (**b**), and Epttn (**c**) of Case_3. Note that the lowercase letters “a–e” represent natural fractures NF_a to NF_e.

Concerning Case_3, its main fracture morphology was similar to Case_2, displaying a right-row echelon feature, with better connections between the newly generated fractures than in Case_2. Additionally, more branch fractures developed around the main fracture (Figure 11a). According to Figure 11b,c, the equivalent shear plastic strain of the main fracture was relatively large, especially in fractures within organic-rich laminae, indicating the shear failure of these fractures. The equivalent plastic tensile strain of fractures that propagated in sandy or tuffaceous laminae was more prominent, suggesting that these fractures mainly experienced tensile failure.

Specifically, the fractures that were initiated from the upper end of NF_e and propagated upward along the direction of NF_e presented a shear–tensile composite failure before entering the organic-rich lamina above the tuffaceous lamina. Their later propagation differed with laminae type, showing tensile fracture in sandy or tuffaceous laminae and shear fracture in organic-rich laminae (Figure 11b,c). The fractures that started from the lower end of NF_e and the upper end of NF_b exhibited composite failure, mainly characterized by tensile strain in the sandy laminae, with shear strain as the main form when entering the organic-rich laminae.

**Figure 12:**
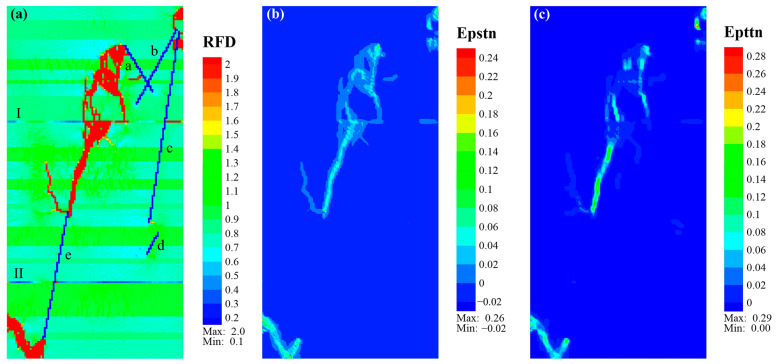
Eventual cloud diagram of *RFD* (**a**), Epstn (**b**), and Epttn (**c**) of Case_4. Note that the lowercase letters “a–e” represent natural fractures NF_a to NF_e and that the numbers “I, II” indicate weak interfaces WI_I and WI_II.

For Case_4, the lower half of the main fracture was analogous to Case_2 and Case_3, but with fewer branch fractures. The upper part was significantly affected by the weak interface I (WI_I), and the fractures were distributed like a network (Figure 12a). The equivalent plastic shear strain was prevalent around all the new fractures in Case_4 (Figure 12b). In contrast, the equivalent plastic tensile strain was mainly concentrated in fractures between the upper end of NF_e and the tuffaceous lamina below WI_I, and around the near-vertical fractures in sandy or tuffaceous laminae above WI_I (Figure 12c). The fractures that initiated from the lower end of NF_e exhibited a tensile–shear composite failure, with advantageous modes differing in different types. In the moderate sandy laminae, tensile fracture was dominant, while in the organic-rich laminae, shear fracture was the main form. For fractures that initiated from the upper end of NF_b, it was similar to Case_3, exhibiting a composite failure dominated by tensile fracture and accompanied by shear fracture.

## 4. Discussion

### 4.1. Influence of Natural Fractures and Laminae on Fracture Propagation and Failure Mode

Shale with different geological structures exhibits similarities in fracture propagation patterns. In all simulated shale models, new fractures were primarily initiated from the ends of existing natural fractures, predominantly between the upper ends of NF_e and NF_a. Some fractures were initiated from the upper ends of NF_b and NF_c, but their subsequent expansion was limited. The lower end of NF_e exhibited fractures uniformly extending toward the bottom boundary before propagating toward the left boundary. Notably, no visible new fractures occurred at both ends of NF_d, resembling the response observed in unconnected natural fractures simulated by Sesetty and Ghassemi [36].

**Figure 13:**
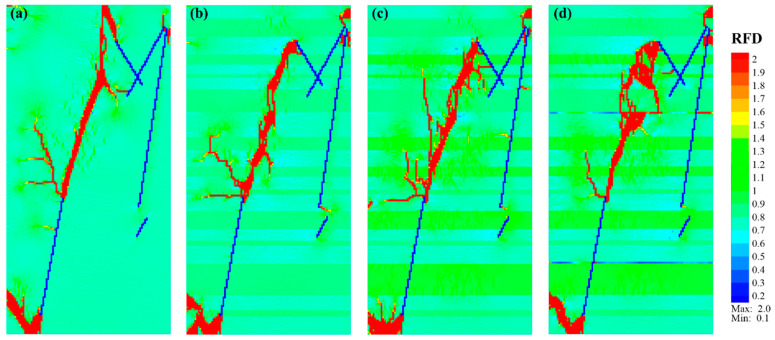
Modeled *RFD* cloud diagrams of shale with various structures under uniaxial compression conditions. (**a**–**d**) Correspond to Case_1, Case_2, Case_3, and Case_4, respectively.

Despite the similarities, the fracture propagation in different models displayed unique characteristics due to variations in lamina type and weak interface. In Case_1, with a relatively simple lamina structure, newly formed fractures tended to follow the direction of natural fractures, forming a nearly straight main fracture with an angle of *β* ≈ 19° with fewer branch fractures (Figure 13a). This mechanism is consistent with one of the propagation mechanisms revealed by Zhang et al. [52], emphasizing propagation along the principle direction of natural fracture. For Case_2, fractures nearly vertically (*β* ≈ 0°) crossed sandy laminae (*β* ≈ 0°), with near-horizontal propagation or in a direction of *β* ≈ 20°–40° in organic-rich laminae (Figure 13b). The fractures expanded in an echelon pattern toward the upper and lower ends, intersecting to form a sausage-like main fracture with *β* ≈ 21°. This fracture path was similar to the universal structure of shear ruptures represented by an echelon of blocks (or slabs) separated by tensile cracks—known as a “domino” structure [53]. Zhao et al. [24] also observed shear failure through the laminae based on compression tests on laminated shale under low confining pressure conditions. Nevertheless, the fracture height of Case_2 was smaller than that of Case_1, similar to the significant inhibitory effects of beddings on fracture height growth revealed by Han et al. [39].

In Case_3, the occurrence of vertically penetrating fractures was more pronounced, potentially due to the presence of tuffaceous laminae enhancing overall brittleness. This phenomenon is similar to fractures propagating along the maximum principle stress direction [52]. Compared to Case_2, more and longer penetrating fractures were visible in Case_3, and the main fracture (*β* ≈ 23°) was distributed in an echelon pattern with numerous secondary fractures (Figure 13c). As for Case_4, fractures extending from the upper end of NF_e reached the vicinity of WI_I, propagating with fractures that vertically crossed and obliquely cut organic-rich laminae before arresting at the weak interface. Fractures extending from the upper end of NF_a were also connected to the weak interface, generating horizontal fractures extending toward both ends and forming a network-like fracture shape (Figure 13d). Zou et al. [26] also pointed out that the coexistence of multiple oblique laminae, microfractures, lithologic interbeds, and lateral mineral composition variations can significantly cause fracture complexity and uncertainty in continental shale. Fracture geometry shapes like “╪” tended to be created due to the intersection of hydraulic fractures with dense horizontal laminae.

All shale models from Case_1 to Case_4 exhibited both shear and tensile failure, with tensile failure prevailing and shear failure occurring around the ends of most natural fractures. Fractures in sandy or tuffaceous laminae tended to propagate parallel or nearly parallel to the maximum compressive stress and presented with tensile fractures, while fractures in the organic-rich laminae primarily exhibited shear fractures with a certain angle to the maximum compressive stress. Specifically, the main fracture in Case_1 was dominated by shear fractures, with local branch fractures primarily displaying tensile fractures. In Case_2, influenced by sandy laminae, tensile fractures and shear fractures were alternately distributed, with relatively clearer tensile fractures. In Case_3, shear fractures were the predominant pattern, with localized echelon tensile fractures distributed in the medium-to-hard laminae. Regarding Case_4, the overall distribution of shear fractures was relatively larger, but clearer tensile fractures were observed within some local laminae, especially in the lower half of the main fracture. This observation aligns with the findings that most shear fractures in hydraulic fractures were natural fractures, while new fractures tended to be tensile fractures [54]. 

### 4.2. Influence of Natural Fractures and Laminae on Fracture Complexity

**Figure 14:**
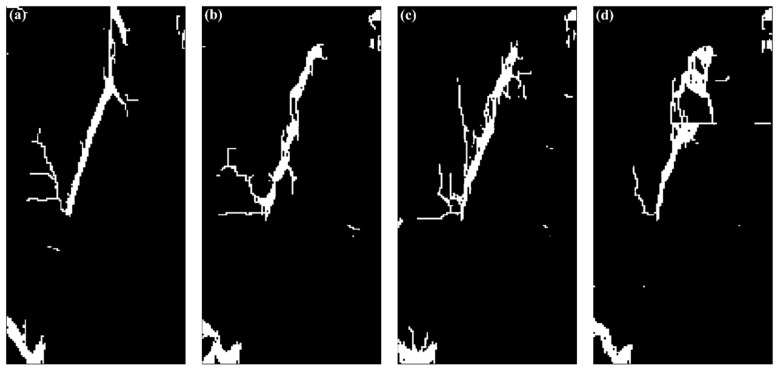
Binary images of modeled *RFD* cloud diagrams of shale with various structures under uniaxial compression conditions. (**a**–**d**) Correspond to Case_1, Case_2, Case_3, and Case_4, respectively. Note that white represents a fractured area, and black indicates an undamaged area.

To quantitatively analyze the fracture complexity of shale with different structures, the fracture area of the final *RFD* image (Figure 13) of each model was quantified. The *RFD* images were processed using the software Image-Pro Plus 6.0 (Trial version). Specifically, they were first segmented and then converted into a binary image, as shown in Figure 14. The white represents the fractured area, and the black indicates the undamaged area. Each model developed an interconnected main fracture area in the middle, and the remaining fractures were independently distributed near the model boundary with a relatively small area. Therefore, statistical analysis was conducted on both the total fracture area and the main fracture area for better comparison. The fracture area of each model was calculated based on the pixels of the fractured region and image resolution.

**Figure 15:**
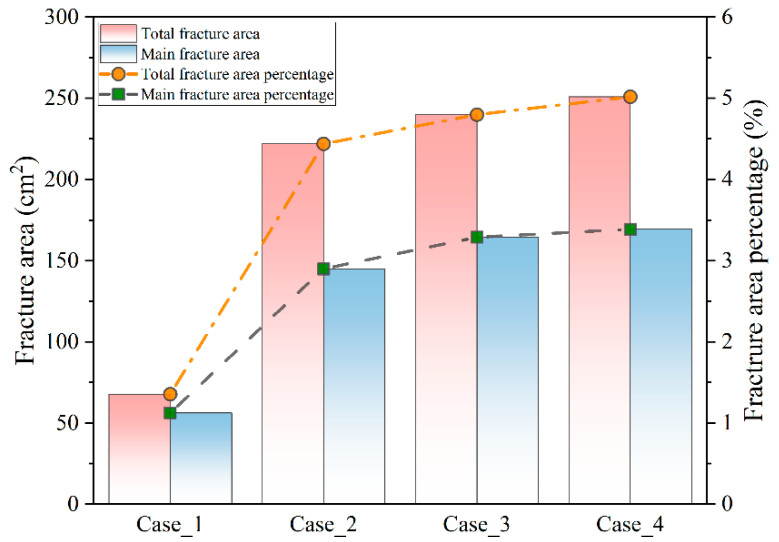
Total fracture area and main fracture area distribution of the shale model with various structures under uniaxial compression conditions.

The statistical fracture areas of various shale models are presented in Figure 15. In Case_1, which consisted of five natural fractures and organic-rich laminae, it exhibited the smallest total fracture area (~67.78 cm^2^) and main fracture area (~56.20 cm^2^), accounting for approximately 1.36% and 1.12% of the total shale model area (5000 cm^2^), respectively. Upon introducing sandy laminae into the shale model in Case_2, there was a significant increase in both the total fracture area (up to 221.91 cm^2^) and the main fracture area (~144.89 cm^2^), accounting for approximately 4.44% and 2.90% of the total model area, respectively.

With the occurrence of tuffaceous laminae in Case_3, the total fracture area increased to 239.85 cm^2^, constituting approximately 4.80% of the total model area. The area of the main fracture also increased to 164.38 cm^2^, with a percentage of 3.29%. Subsequently, when weak interfaces occurred between laminae in Case_4, the total fracture area slightly increased to approximately 250.88 cm^2^, representing 5.02% of the total model area. The main fracture area was slightly larger than that of Case_3, with a value of about 169.29 cm^2^, accounting for 3.39% of the total model area.

As medium-to-hard laminae (such as sandy laminae and tuffaceous laminae) and weak interfaces successively occurred, the shale structure became more complex. The proportions of the total fracture area and the main fracture area increased by up to 270.12% and 201.24%, respectively. This indicates that the lamina types and weak interfaces play a crucial role in shaping the fracture morphology of continental shale under uniaxial compression conditions. Medium-to-hard laminae are helpful in generating more tensile fractures. The presence of weak interfaces contributed to the formation of network fractures. Both of them are beneficial to enhance the fracture complexity. However, the role of beddings in fracture networks is controversial. The comparison of hydraulic simulations on four cubic models (rock matrix model, bedding model, joint model, and bedding and joint model) suggested that preexisting discontinuities imposed a barrier for fracture growth in most cases, resulting in a smaller fracture area and higher fluid pressure for fracture extension [13]. Yang et al. [23] pointed out that the bedding structure of continental shale probably did not participate in the formation of complex fracture networks during triaxial compression tests. This contradiction is probably due to the fact that the rock matrixes in the cubic models and tested shales were identical while we changed the laminae in our models from one type to three types to resemble the authentic laminae developed in continental shale.

This research provides a numerical reference for understanding the combined effect of natural fractures, various laminae, and weak interfaces on fracture propagation and fracture mode of continental shale. The fracture area in this study is analogous to the stimulated reservoir volume (SRV) during hydraulic fracturing to some extent. A larger fracture area suggests a more complex fracture shape, implying a larger SRV. It has been demonstrated in engineering practice that interlayered shale reservoirs exhibit superior well productivity compared to pure shale reservoirs [55,56]. The modeling results provide valuable insight into the different performances of shale reservoirs with various rock mass structures. The results can be applied in SRV optimization for shale oil and gas exploration by integrating natural fractures, lamina types, and weak interfaces in continental shale. Three-dimensional simulations considering various geological parameters and fracturing parameters (such as stress difference, fluid viscosity, injection rate, etc.) should be conducted in further research to promote engineering practicability due to the complex geological conditions of continental shale reservoirs. These simulations should take into account the numerous other factors that influence fracturing response, such as porosity, organic matter maturity, pressure coefficient, gas/oil ratio, brittle mineral content, etc.

## 5. Conclusions

Based on the structural characteristics of continental shale, four different shale models developed with natural fractures, soft-to-hard laminae, and weak interfaces (Case_1 to Case_4) were generated and simulated using CASRock. The mechanical behavior of various continental shale models under uniaxial compression conditions can be summarized as follows:

The uniaxial compressive strength of shale models increased with the occurrence of soft-to-hard laminae by up to 34.48%, from 3.98 MPa to 5.57 MPa. It then decreased with weak interfaces by up to 18.97% (4.51 MPa). The elastic modulus, on the other hand, remained relatively constant, approximately in the range of 13.52–14.38 GPa.

Fracture initiation occurred exclusively at the ends of pre-existing natural fractures. New fractures propagated obliquely with an angle of *β* ≈ 20°–30° relative to the direction of compressive stress in soft laminae. In medium-to-hard laminae, they shifted to propagate almost parallel to the compressive stress (*β* ≈ 0°).

The newly generated main fracture changed from a nearly linear pattern to an echelon pattern with more secondary fractures and a network-like morphology with successive occurrences of soft-to-hard laminae and weak interfaces. The failure mode changed from shear failure to combined tensile–shear fracture, and ultimately to shear fracture with localized echelon tensile fractures.

The ultimate total fracture area increased continuously as the shale structure became more complex, reaching a maximum increase of 270.12%. The coexistence of natural fractures, soft-to-hard laminae, and weak interfaces helps increase fracture complexity. The findings provide a numerical reference for optimizing the stimulated reservoir volume of shale by taking into account its genuine structure features.

## Figures and Tables

**Table 1:** Input parameters for various materials in shale models.

Material Type	Mechanical Parameter							
	*E* (GPa)	*v*	*c*_0_ (MPa)	*c_r_* (MPa)	*φ*_0_ (°)	*φ_r_* (°)	*τ*_0_ (MPa)	*τ_r_* (MPa)
Organic-rich lamina	15	0.3	6	0.6	35	20	2	0.1
Sandy lamina	43	0.3	22.5	2.25	40	30	6	0.35
Tuffaceous lamina	120	0.2	29	1	42	30	3	0.1
Weak interface	5	0.3	0.25	0.01	30	20	0.2	0.01
Natural fracture	0	0.3	1	0.1	30	20	0.5	0.1

## Data Availability

The datasets of this study are available from the corresponding author upon reasonable request and within the framework of scientific research projects.
